# Activity guided isolation and modification of juglone from *Juglans regia* as potent cytotoxic agent against lung cancer cell lines

**DOI:** 10.1186/s12906-015-0920-0

**Published:** 2015-11-03

**Authors:** Xue-Bang Zhang, Chang-Lin Zou, Yu-Xia Duan, Fang Wu, Gang Li

**Affiliations:** Department of Chemoradiation Oncology, The First Affiliated Hospital of Wenzhou Medical University, Wenzhou, 325000 China; Department of Radiation, The First Affiliated Hospital of Wenzhou Medical University, Wenzhou, 325000 China; Department of Gastroenterology, The First Affiliated Hospital of Wenzhou Medical University, Wenzhou, 32500 China

## Abstract

**Background:**

*Juglans regia* has been found to exhibit significant anticancer activity against various human cancer cell lines. This study was undertaken to isolate the active chemical constituent (Juglone) and to investigate its cytotoxic activity along with its various analogs against different human cancer cell lines.

**Methods:**

Isolation of juglone, a napthoquinone, from the chloroform extract of the root part of *Juglans regia* was executed by flash chromatography using silica gel as stationary phase. The isolated Juglone was used as starting material for the further synthesis of a novel series of triazolyl analogs using click chemistry approach to investigate their cytotoxic potential against different human cancer cell lines using 3-(4,5-Dimethylthiazol-yl)-diphenyl tetrazoliumbromide (MTT) assay.

**Results:**

The different extracts of *Juglans regia* and the isolated compound (juglone) exhibited satisfactory cytotoxic activity against a panel of eight different human cancer cell lines namely, prostate colon (Colo-205 and HCT-116), breast (T47D), prostate (PC-3 and DU-145), skin (A-431) and lung (NCI-H322 and A549). Interestingly, all the synthesised analogs displayed enhanced and selective cytotoxic activity against lung cancer cell lines only. Of the synthesized derivatives, **15a** and **16a** displayed the best activity with IC_50_ of 4.72 and 4.67 μM against A549 cells. Both these derivatives exhibited superior potency to BEZ-235 against both the lung cancer cell lines. So far as the structural aspects are concerned, electron withdrawing substituents at the ortho position of R moiety of the triazolyl analogs seem to be essential for attaining better activity.

**Conclusion:**

The present study demonstrates the selective and enhanced cytotoxic activity of the triazolyl analogs of juglone against NCI-H322 and A549 human lung cancer cell lines. Some derivatives exhibited superior potency to BEZ-235, a commercially available anticancer agent.

## Background

*Juglans regia* Linn. also known as Persian walnut, white walnut, English walnut or common walnut, belongs to the family juglandaceae. It is widely distributed in southern Europe, northern Africa, eastern Asia, the USA and western South America [1]. China is the leading world producer of walnut, followed by the USA, Iran, Turkey, Ukraine, Romania, France and India [2]. *Juglans regia* leaves have been used worldwide in traditional system of medicine as antimicrobial, antihelmintic, astringent, antidiarrhoeal, hypoglycaemic, depurative, tonic, carminative, and for the treatment of sinusitis, cold and stomach ache [3, 4]. In Turkish folk medicine, its leaves are used to reduce fever and to alleviate the rheumatic pain [5, 6]. The kernel of *J. regia* has been used for the treatment of inflammatory bowel disease in Iranian traditional medicine [7]. The bark, branches and exocarp of the immature green fruit of this medicinal plant have been used to treat gastric, liver and lung cancer in China [8, 9]. The herbal preparations of walnut (*Juglans*) have been reported in Chinese traditional medicine for the treatment of a host of diseases including cancer [10, 11].

Quinones have been extensively scrutinised for their potential anticancer activities and such research programmes have yielded numerous clinically significant scaffolds like doxorubicin, mitoxanthrone, siantropin etc. However, due to the prevalence of drug resistance many more anticancer agents are still under scrutiny [12, 13]. Juglone (5-hydroxy-1,4-naphthaquinone) is one such pharmacologically active napthoquinone isolated from various *Juglans* (family: Juglandaceae) species. Previous studies have demonstrated the potential anticancer activity of juglone in various in vivo tumour models [14–18]. In addition to this, recent studies have shown the significant cytotoxic activity of juglone against various cancer cell lines [19, 20]. The cytotoxic activity of juglone is primarily attributed to induction of reactive oxygen species which leads to an altered redox homeostasis in the cell causing apoptotic as well as necrotic cell death. Additionally, juglone is believed to inhibit Pin1 (Peptidyl-prolyl isomerase), known to be over-expressed in many cancer types and has been hypothesized to be a chemotherapeutic drug target [21–25]. Juglone has also been reported to exert certain toxic effects to normal tissues causing acute irritant contact dermatitis [25].

Despite the excellent anticancer potential, quinones present high lipophilicity. The high lipophilicity facilitates easy permeation through biomembranes thereby enhancing their cytotoxicity in vitro, but the higher lipophilicity considerably reduces their bioavailability in vivo. The cellular delivery of anticancer molecules is an important aspect in the field of anticancer therapy and several means have been employed for improving the bioavailability as well as enhancing the activity and reducing their toxicity. These limitations of the anticancer chemotherapies could be overcome by introduction of hydrogen bond donors like N and O, thereby enhancing the aqueous solubility, improved pharmacokinetic potential and therefore improved bioavailability. In this connection, the concept of click chemistry inspired synthesis of triazolyl analogs seems to be a sensible path, wherein three nitrogen atoms are incorporated in a five membered heterocyclic ring.

Click chemistry [26] has recently attracted great attention from the medicinal chemists primarily in the field of triazolyl analog synthesis of natural products including alkaloids [27, 28], coumarins [29, 30], saponins [31], steroids [32] and triterpenes [33]. Triazole derivatives have a great reputation in medicinal chemistry and have been successfully used as antiviral, antibacterial, antifungal, anti-tuberculosis, anticonvulsant, antidepressant, anti-inflammatory and anticancer agents [34, 35]. Anastrozole, letrozole and vorozole are some important examples of clinically significant triazole derived antineoplastic drugs [35]. Therefore, the design and synthesis of natural product derived triazolyl analogs is the prospective path for the development of novel antineoplastic agents with better activity, lower toxicity as well as higher selectivity.

Inspired by above cited literature and the potential anticancer activity of triazoles in general and juglone in particular, we designed this work towards the synthesis of a diverse series of novel triazolyl analogs of biological interest using juglone (**1**) as an important starting material. All the triazolyl analogs along with different extracts of *Juglans regia* were subjected to MTT [3-(4,5-Dimethylthiazol-yl)-diphenyl tetrazoliumbromide] cytotoxicity assay against a panel of eight different human cancer cell lines namely, prostate colon (Colo-205 and HCT-116), breast (T47D), prostate (PC-3 and DU-145), skin (A-431) and lung (NCI-H322 and A549) to check their cytotoxic potential. To the best of our knowledge, there are no reports on the synthesis of triazolyl analogs of juglone and their bioevaluation. This work provides the initial report on structure-activity relationship of triazolyl analogs of juglone with the aim to prepare analogs with enhanced bioavailability, lesser toxicity, better activity and improved selectivity. This strategy would provide an important step towards the rationalisation of lead properties of juglone.

## Methods

All the solvents and reagents for the preparation of extracts, chemical synthesis and biological assays were obtained from Sigma Aldrich. The chemical reactions were monitored by using F254 silica gel TLC plates (E. Merck) using ceric ammonium sulphate and UV torch for the detection of spots. All the compounds were purified by column chromatography on silica gel (60–120 mesh). ^1^H NMR and ^13^C NMR spectra (with chemical shifts expressed in *δ* and coupling constants in Hertz) were recorded on Bruker DPX 400 instrument using CDCl_3_ as the solvent with TMS as internal standard. High resolution mass spectra (HRMS) were recorded on Agilent Technologies 6540 instrument and IR recorded on an FT-IR Bruker (270–30) spectrophotometer. Melting points of compounds were recorded on Buchi melting point apparatus.

### Plant materials

The bark of *Juglans regia* was collected from Wenzhou, China and the dried voucher specimen (No. JR 0014) was deposited in the herbarium of the Wenzhou Medical University, China.

### Preparation of plant extracts

The air-dried chopped plant material was extracted with different organic solvents including hexane, chloroform, ethyl acetate and methanol separately at room temperature for 24 h (three times). These extracts were then concentrated under reduced pressure at 35 °C using a rotary evaporator (Buchi, Switzerland).

### Isolation of juglone

Juglone was isolated in good amount from the root parts of *Juglans regia* (duly authenticated by the taxonomist). Juglone was isolated through repeated column chromatography over silica gel 60–120 mesh from the chloroform extract and characterized by spectral data analysis and by comparing with the authentic sample.

### Synthesis of 2 (Scheme 1)

**Scheme 1:**
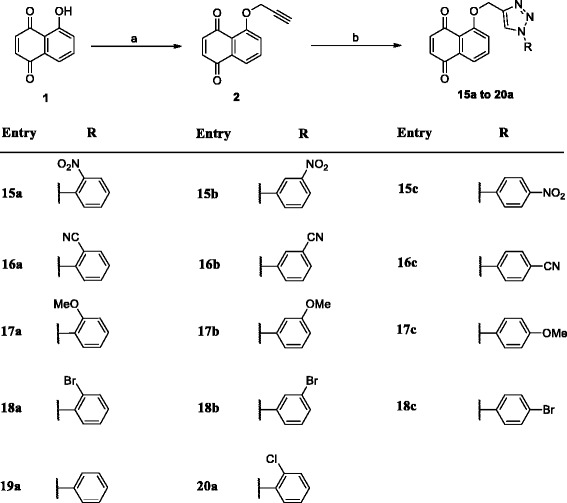
Reagents and conditions: (a) Propargyl bromide, NaH, acetonitrile, rt, 3 h (b) N_3_R, CuSO_4_.5H_2_O, sodium ascorbate, t-butanol:H_2_O (1:1), rt, 30 min

To a solution of juglone (**1**) (2 g, 11.49 mmol) in acetonitrile (10 ml), NaH (530 mg, 22.98 mmol) was added portionwise and stirred for 10 min at 20 °C. Propargyl bromide (1.5 ml, 15 mmol) was then added drop wise and the suspension was stirred for 2 h. Progress of reaction was monitored using TLC at regular intervals. After the completion of reaction, the reaction mixture was extracted with ethyl acetate (3x30 ml) which on subsequent purification over silica gel column resulted in the isolation of pure product **2** in 67 % yield. Colourless solid, mp: 125 °C. ^1^H NMR (CDCl_3_, 400 MHz) *δ*: 7.78 (m, 2H), 7.28 (d, *J* = 8.1 Hz, 1H), 6.95 (m, 2H), 4.43 (s, 2H), 2.50 (s, 1H). ^13^C NMR (126 MHz, CDCl_3_) *δ*: 190.53, 184.50, 161.70, 139.84, 138.89, 136.80, 132.01, 124.74, 119.40, 115.22, 79.20, 76.54, 58.80. HR-ESIMS *m/z*: calculated for C_13_H_8_O_3_ [M + H]^+^ 213.0553, found 213.0595.

### General procedure for synthesis of azides

The azides were prepared as per the known method [29]. To a solution of particular aromatic amine in 1,4-Dioxane (at the rate of 50 mg/ml) at −15.0 °C, 5 equivalents of 2 M Sulphuric acid was added in small instalments while stirring. After 5 min 2 equivalents of 3 M sodium nitrite was added drop wise and after 30 min 3 equivalents of 3 M sodium azide was added drop wise carefully. Reaction was brought to room temperature and extracted with diethyl ether for at least three times. Organic layers were washed with saturated sodium bicarbonate solution two times, dried over anhydrous sodium sulphate and concentrated to a minimum volume under reduced pressure on rotavapour.

### General procedure for synthesis triazolyl derivatives (15a to 20a)

Compound **2** (3 mmol) and different freshly prepared organic azides (3 mmol) were sonicated in 10 ml of a 2:1 water:*t*-butanol mixture with sodium ascorbate (0.3 mmol, 300 μL of freshly prepared 1 M solution in water) followed by copper (II) sulfate pentahydrate (0.03 mmol, in 100 μl of water). The reaction mixture was extracted with ethyl acetate (30x3 ml) and the combined organic layer was dried over sodium sulphate and purified through column chromatography to give pure products in excellent yields of 85–90 %.

#### 5-((1-(2-Nitrophenyl)-1H-1,2,3-triazol-4-yl)methoxy)juglone **15a**

Yellow liquid; Yield: 87 %; ^1^H NMR (400 MHz, CDCl_3_) *δ*: ^1^H NMR (CDCl_3_, 400 MHz) *δ*: 8.07 (d, *J* = 8.0 Hz, 1H), 7.96 (s, 1H), 7.80 (t, *J* = 8.0 Hz, 1H), 7.75 (m, 2H), 7.69 (m, 2H), 7.25 (d, *J* = 8.0 Hz, 1H), 6.92 (2H, m), 4.40 (s, 2H). ^13^C NMR (126 MHz, CDCl_3_) *δ*: 191.02, 185.10, 160.77, 146.47, 144.41, 139.84, 138.89, 136.80, 133.80, 132.01, 130.63, 130.37, 127.62, 125.45, 124.74, 124.21, 119.40, 115.22, 65.25. HR-ESIMS *m/z*: calculated for C_19_H_12_N_4_O_5_ [M + H]^+^ 377.0816, found 377.0859.

#### 5-((1-(3-Nitrophenyl)-1H-1,2,3-triazol-4-yl)methoxy)juglone **15b**

Yellowish liquid; Yield: 88 %; ^1^H NMR (400 MHz, CDCl_3_) *δ*: 8.60 (s, 1H), 8.30 (m, 1H), 8.26 (s, 1H), 8.18 (m, 1H), 7.75 (m, 3H), 7.30 (d, *J* = 8.0 Hz, 1H), 6.98 (m, 2H), 4.24 (s, 2H). ^13^C NMR (126 MHz, CDCl_3_) *δ*: 190.88, 185.21, 161.72, 148.99, 140.11, 138.92, 137.86, 136.97, 132.33, 131.01, 125.84, 124.74, 123.08, 120.59, 120.59, 120.11, 115.62, 115.01, 65.44. HR-ESIMS *m/z*: calculated for C_19_H_12_N_4_O_5_ [M + H]^+^ 377.0816, found 377.0863.

#### 5-((1-(4-Nitrophenyl)-1H-1,2,3-triazol-4-yl)methoxy)juglone **15c**

Yellow liquid; Yield: 88 %; ^1^H NMR (400 MHz, CDCl_3_) *δ*: 8.44 (d, *J* = 9.0 Hz, 2H), 8.29 (s, 1H), 7.98 (d, *J* = 9.0 Hz, 2H), 7.81 (m, 2H), 7.29 (d, *J* = 8.0 Hz, 1H), 6.97 (2H, m), 4.29 (s, 2H). ^13^C NMR (126 MHz, CDCl_3_) *δ*: 190.60, 184.34, 161.65, 147.61, 147.12, 141.24, 139.91, 138.88, 136.80, 132.05, 125.58, 125.58, 124.04, 120.56, 120.30, 120.30, 119.41, 115.02, 59.81. HR-ESIMS *m/z*: calculated for C_19_H_12_N_4_O_5_ [M + H]^+^ 377.0816, found 377.0861.

#### 5-((1-(2-Cyanophenyl)-1H-1,2,3-triazol-4-yl)methoxy)juglone **16a**

Yellowish liquid; Yield: 89 %; ^1^H NMR (400 MHz, CDCl_3_) *δ*: 8.35 (s, 1H), 7.86 (m, 3H), 7.80 (m, 2H), 7.63 (m, 1H), 7.30 (d, *J* = 8.0 Hz, 1H), 6.97 (2H, m), 4.23 (s, 2H). ^13^C NMR (126 MHz, CDCl_3_) *δ*: 190.62, 184.44, 161.68, 146.69, 140.10, 138.92, 138.68, 136.82, 134.39, 134.39, 132.06, 129.46, 124.66, 125.37, 123.15, 119.49, 115.70, 115.20, 65.12. HR-ESIMS *m/z*: calculated for C_20_H_12_N_4_O_3_ [M + H]^+^ 357.0917, found 357.1002.

#### 5-((1-(3-Cyanophenyl)-1H-1,2,3-triazol-4-yl)methoxy)juglone **16b**

Yellow liquid; Yield: 85 %; ^1^H NMR (400 MHz, CDCl_3_) *δ*: 8.35 (m, 1H), 8.25 (s, 1H), 8.16 (m, 1H), 7.80 (m, 2H), 7.65 (m, 1H), 7.68 (t, *J* = 8.2 Hz, 1H), 7.29 (d, *J* = 8.0 Hz, 1H), 6.92 (m, 2H), 4.25 (s, 2H). ^13^C NMR (126 MHz, CDCl_3_) *δ*: 190.65, 184.64, 162.04, 147.10, 146.61, 141.20, 139.92, 138.87, 136.57, 132.00, 125.51, 125.51, 124.77, 120.55, 120.25, 119.99, 114.82, 120.25, 65.31. HR-ESIMS *m/z*: calculated for C_20_H_12_N_4_O_3_ [M + H]^+^ 357.0917, found 357.0989.

#### 5-((1-(4-Cyanophenyl)-1H-1,2,3-triazol-4-yl)methoxy)juglone **16c**

Yellow liquid; Yield: 95 %; ^1^H NMR (400 MHz, CDCl_3_) *δ*: 8.20 (s, 1H), 7.92 (d, *J* = 8.8 Hz, 2H), 7.86 (d, *J* = 8.8 Hz, 2H), 7.82 (m, 2H), 7.34 (d, *J* = 8.0 Hz, 1H), 6.99 (m, 2H), 4.23 (s, 2H). ^13^C NMR (126 MHz, CDCl_3_) *δ*: 191.09, 185.21, 161.88, 147.48, 140.47, 139.95, 133.95, 138.99, 136.97, 133.95, 131.88, 124.52, 120.43, 120.43, 120.40, 120.02, 117.71, 115.77, 65.36. HR-ESIMS *m/z*: calculated for C_20_H_12_N_4_O_3_ [M + H]^+^ 357.0917, found 357.1003.

#### 5-((1-(2-Methoxyphenyl)-1H-1,2,3-triazol-4-yl)methoxy)juglone **17a**

Yellow liquid; Yield: 89 %; ^1^H NMR (400 MHz, CDCl_3_) *δ*: 8.2 (s, 1H), 7.80 (m, 2H), 7.77 (d, *J* = 8.0 Hz, 1H), 7.42 (m, 1H), 7.29 (d, *J* = 8.0 Hz, 1H), 7.10 (m, 2H), 6.93 (m, 2H), 4.22 (s, 2H), 3.60 (s, 3H). ^13^C NMR (126 MHz, CDCl_3_) *δ*: 191.08, 184.82, 162.34, 151.10, 139.98, 138.73, 136.49, 131.71, 130.05, 126.33, 125.68, 125.52, 125.42, 121.35, 121.21, 119.88, 115.38, 112.18, 65.32, 55.60. HR-ESIMS *m/z*: calculated for C_20_H_15_N_3_O_4_ [M + H]^+^ 362.1071, found 362.1133.

#### 5-((1-(3-Methoxyphenyl)-1H-1,2,3-triazol-4-yl)methoxy)juglone **17b**

Yellow liquid; Yield: 90 %; ^1^H NMR (400 MHz, CDCl_3_) *δ*: 8.10 (s, 1H), 7.74 (m, 2H), 7.38 (m, 2H), 7.25 (m, 1H), 7.22 (d, *J* = 8.0 Hz, 1H), 6.93 (m, 2H), 6.97 (m, 1H), 4.23 (s, 2H), 3.62 (s, 3H). ^13^C NMR (126 MHz, CDCl_3_) *δ*: 189.92, 183.80, 161.34, 145.96, 139.25, 137.91, 137.12, 136.22, 132.05, 130.49, 124.44, 120.91, 119.76, 119.04, 115.18, 114.49, 112.29, 106.21, 65.41, 55.62. HR-ESIMS *m/z*: calculated for C_20_H_15_N_3_O_4_ [M + H]^+^ 362.1071, found 362.1129.

#### 5-((1-(4-Methoxyphenyl)-1H-1,2,3-triazol-4-yl)methoxy)juglone **17c**

Yellow liquid; Yield: 88 %; ^1^H NMR (400 MHz, CDCl_3_) *δ*: 8.05 (s, 1H), 7.79 (m, 2H), 7.66 (d, *J* = 8.8 Hz, 2H), 7.28 (d, *J* = 8.0 Hz, 1H), 7.05 (d, *J* = 8.8 Hz, 2H), 6.97 (m, 2H), 4.59 (s, 2H), 3.88 (s, 3H). ^13^C NMR (126 MHz, CDCl_3_) *δ*: 190.75, 184.88, 161.37, 159.77, 146.19, 139.83, 138.19, 136.77, 132.14, 130.53, 124.91, 122.21, 122.21, 121.18, 119.53, 115.31, 114.74, 114.74, 65.42, 55.62. HR-ESIMS *m/z*: calculated for C_20_H_15_N_3_O_4_ [M + H]^+^ 362.1071, found 362.1131.

#### 5-((1-(2-Bromophenyl)-1H-1,2,3-triazol-4-yl)methoxy)juglone **18a**

Yellowish liquid; Yield: 88 %; ^1^H NMR (400 MHz, CDCl_3_) *δ*: 8.07 (s, 1H), 7.77 (m, 3H), 7.56 (m, 1H), 7.49 (m, 1H), 7.39 (m, 1H), 7.33 (d, *J* = 8.0 Hz, 1H), 7.03 (m, 2H), 4.25 (s, 2H). ^13^C NMR (126 MHz, CDCl_3_) *δ*: 190.88, 184.72, 161.70, 145.28, 140.20, 138.92, 136.83, 136.71, 133.94, 132.19, 131.07, 128.48, 128.20, 124.73, 124.74, 119.40, 118.48, 115.22, 65.35. HR-ESIMS *m/z*: calculated for C_19_H_12_BrN_3_O_3_ [M + H]^+^ 410.0070; [M + 2]^+^ 411.0042, found 410.0182, 411.0138.

#### 5-((1-(3-Bromophenyl)-1H-1,2,3-triazol-4-yl)methoxy)juglone **18b**

Yellow liquid; Yield: 88 %; ^1^H NMR (400 MHz, CDCl_3_) *δ*: 8.06 (s, 1H), 7.81 (m, 2H), 7.42 (m, 2H), 7.30 (m, 1H), 7.18 (d, *J* = 8.0 Hz, 1H), 6.95 (m, 2H), 7.01 (m, 1H), 4.30 (s, 2H). ^13^C NMR (126 MHz, CDCl_3_) *δ*: 190.12, 183.38, 161.52, 145.10, 138.71, 137.08, 137.15, 136.25, 132.12, 130.55, 125.02, 120.18, 119.55, 118.82, 115.51, 114.76, 112.30, 106.15, 65.77. HR-ESIMS *m/z*: calculated for C_19_H_12_BrN_3_O_3_ [M + H]^+^ 410.0070, [M + 2]^+^ 411.0042, found 410.0179, 411.0134.

#### 5-((1-(3-Bromophenyl)-1H-1,2,3-triazol-4-yl)methoxy)juglone **18c**

Yellow liquid; Yield: 89 %; ^1^H NMR (400 MHz, CDCl_3_) *δ*: 8.09 (s, 1H), 7.82 (m, 2H), 7.67 (d, *J* = 9.1 Hz, 2H), 7.63 (d, *J* = 9.1 Hz, 2H), 7.31 (d, *J* = 8.0 Hz, 1H), 6.92 (m, 2H), 4.23 (s, 2H). ^13^C NMR (126 MHz, CDCl_3_) *δ*: 190.89, 184.65, 161.70, 146.15, 139.92, 138.98, 137.15, 135.12, 132.02, 131.91, 131.91, 124.25, 121.28, 120.80, 120.80, 119.59, 119.53, 115.22, 64.40. HR-ESIMS *m/z*: calculated for C_19_H_12_BrN_3_O_3_ [M + H]^+^ 410.0070, [M + 2]^+^ 411.0042, found 410.0180, 411.0140.

#### 5-((1-Phenyl-1H-1,2,3-triazol-4-yl)methoxy)juglone **19a**

Yellow liquid; Yield: 90 %; ^1^H NMR (400 MHz, CDCl_3_) *δ*: 8.10 (s, 1H), 7.76 (m, 4H), 7.54 (m, 2H), 7.44 (m, 1H), 7.29 (d, *J* = 8.0 Hz, 1H), 6.99 (m, 2H), 4.24 (s, 2H). ^13^C NMR (126 MHz, CDCl_3_) *δ*: 190.90, 184.45, 161.79, 146.50, 139.98, 138.92, 137.19, 136.86, 132.35, 129.75, 129.75, 128.68, 124.74, 120.83, 120.48, 120.48, 119.40, 115.27, 65.55. HR-ESIMS *m/z*: calculated for C_19_H_13_N_3_O_3_ [M + H]^+^ 332.0965, found 332.1018.

#### 5-((2-Chlorophenyl-1H-1,2,3-triazol-4-yl)methoxy)juglone **20a**

Yellow liquid; Yield: 89 %; ^1^H NMR (400 MHz, CDCl_3_) *δ*: 8.10 (s, 1H), 7.76 (m, 3H), 7.42 (m, 1H), 7.35 (d, *J* = 8.0 Hz, 1H), 7.12 (m, 2H), 6.95 (m, 2H), 4.22 (s, 2H). ^13^C NMR (126 MHz, CDCl_3_) *δ*: 190.98, 185.04, 161.68, 145.48, 140.04, 138.92, 136.90, 134.98, 132.12, 130.79, 130.67, 128.46, 127.94, 127.75, 124.69, 124.38, 119.14, 115.18, 65.32. HR-ESIMS *m/z*: calculated for C_19_H_12_ClN_3_O_3_ [M + H]^+^ 366.0575, found 366.0638.

### MTT [3-(4,5-dimethylthiazolyl-2)-2,5-diphenyltetrazolium bromide] assay

All the compounds were evaluated against a panel of eight different human cancer cell lines namely prostate colon (Colo-205 and HCT-116), breast (T47D), prostate (PC-3 and DU-145), skin (A-431) and lung (NCI-H322 and A549) using MTT assay in a 96 well plate. Cells were routinely maintained in RPMI 1640 (Sigma Aldrich) supplemented with 10 % FBS (Merck) and 1 % penicillin G and streptomycin (Sigma Aldrich) at 37 °C in a humidified incubator with 5 % CO_2_ and were subcultured at 1:5 ratio once a week. For antiproliferative activity, compounds were dissolved in cell culture grade DMSO. Briefly, cells (10^4^ cells/well) were cultured in 96 well tissue culture plates and treated with different concentrations of compounds for 48 h. At the end of incubation, 20 μL of MTT (2.5 mg/mL) was added to the wells and incubated for 4 h. Absorbance was recorded at 570 nm using Eliza Plate Reader. Inhibition of formation of coloured MTT formazan was taken as an index of cytotoxicity activity. Further the IC_50_ values on the cancer cells of different tissue origin used for screening were determined by non-linear regression analysis using graph pad prism software [36].

## Results

### Extraction, isolation and preparation of juglone analogs

In this study, the different extracts of *Juglans regia* bark were prepared using hexane, chloroform, ethyl acetate and methanol separately and subjected to cytotoxic activity at 100 μM concentrations. Among these extracts, chloroform extract displayed the best activity at preliminary cytotoxicity screening against prostate colon (Colo-205 and HCT-116), breast (T47D), prostate (PC-3 and DU-145), skin (A-431) and lung (NCI-H322 and A549) cancer cell lines. Chloroform extract was further chromatographed on a flash chromatograph leading to the isolation of the active compound i.e. juglone.

Juglone (**1**) was used as the starting material and was subjected propargylation at the only hydroxyl group with propargyl bromide in presence of NaH in acetonitrile to form 5-prop-2-yn-1-yloxy-juglone (**2**) (Scheme 1). The structure of **2** was confirmed by spectral data analysis. From the elemental analysis and HR-ESIMS (*m/z* 213.0595), this compound was assigned the molecular formula C_13_H_8_O_3_. Proton singlets in ^1^H-NMR at *δ* 2.50 and 4.43 integrating for one and two protons respectively, were assigned to terminal alkyne proton and two methylenic protons of propargylic moiety respectively. The alkylated product (**2**) was allowed to undergo Huisgen 1, 3-dipolar cycloaddition reaction with freshly prepared aromatic azides under sharpless click chemistry conditions [CuSO_4_.5H_2_O and sodium ascorbate in *t*-BuOH:H_2_O (1:1)] to yield regioselectively 1,4-disubstituted-1,2,3-triazoles (**15a** to **20a**) in excellent yields (Scheme 1). A diverse series of triazolyl analogs of juglone was prepared for SAR studies. All the synthesised analogs were characterised by spectral data analysis. The formation of products was confirmed by a triazole proton singlet at around 8.0 ppm in its ^1^H-NMR spectrum besides the other aromatic signals. ^13^C NMR and HR-ESIMS techniques were also employed for the characterisation of products.

### Anti-proliferative activity

All the newly synthesized analogs along with the juglone and different extracts were screened through MTT cytotoxicity assay against a panel of eight human cancer cell lines including prostate colon (Colo-205 and HCT-116), breast (T47D), prostate (PC-3 and DU-145) and skin (A-431) and lung (NCI-H322 and A549). The parent juglone (**1**) and chloroform extract exhibited broad-spectrum cytotoxicity against all the tested cancer cells. Interestingly, at the preliminary screening concentration (50 μM), all the triazolyl anaolgs displayed selective cytotoxicity against lung (NCI-H322 and A549) cancer cell lines in a dose dependent manner. The other cell lines were ineffectively sensitized at the preliminary screening. In order to generate the IC_50_ values, all the derivatives were further assayed at different concentrations (2–50 μM) against NCI-H322 and A549 cell lines (Table 1). The IC_50_ values are the average of triplicate analysis. In this assay the anticancer BEZ-235 was used as a positive control.

**Table 1:** IC_50_ values of analogs against NCI-H322, A549 cancer lines

Entry	Lung (NCI-H322)	Lung (A549)
	IC_50_ (μM)	
Juglone	19.32 ± 0.83	16.70 ± 0.88
15a	8.90 ± 0.20	4.72 ± 0.20
15b	15.60 ± 0.83	12.86 ± 0.48
15c	10.96 ± 0.68	10.20 ± 0.43
16a	7.94 ± 0.34	4.67 ± 0.18
16b	17.22 ± 1.18	15.08 ± 0.92
16c	13.30 ± 0.87	12.95 ± 0.81
17a	29.08 ± 1.20	23.83 ± 1.54
17b	26.52 ± 1.20	20.28 ± 1.15
17c	32.88 ± 1.63	25.32 ± 1.77
18a	24.34 ± 1.28	20.55 ± 0.82
18b	21.82 ± 1.25	19.12 ± 0.89
18c	24.55 ± 1.54	20.26 ± 0.72
19a	18.98 ± 0.99	15.12 ± 0.47
20a	18.28 ± 0.96	12.88 ± 0.42
BEZ-235	9.80 ± 0.25	6.28 ± 0.28

BEZ-235 was used as positive control

Among the synthesized analogs, 15a and 16a bearing an *o*-nitro and *o*-cyanophenyl R moieties respectively, displayed the best activity against both the lung cancer cell lines. 16a exhibited the IC_50_ of 4.67 and 7.94 μM against A549 and NCI-H322 cells respectively, while as, **15a** displayed the IC_50_ of 4.72 and 8.90 μM against A549 and NCI-H322 cells respectively. Both these derivatives displayed better cytotoxic activity than the standard BEZ-235 against A549 and NCI-H322 cells. **17a**, **17b** and**17c** with *o*, *m* and *p*-methoxyphenyl R moieties respectively were weakly active towards both the tested cancer cell lines. Among these derivatives, **17b** with *m*-methoxyphenyl R moiety displayed slightly better activity than the corresponding *o* and *p-*methoxyphenyl counterparts. The analogs **18a**, **18b** and **18c** bearing *o*, *m* and *p*-bromophenyl R moieties were also less active than the parent juglone. Herein, the analog with *m*-bromophenyl R moiety displayed slightly better activity than the corresponding derivatives with *o* and *p*-bromophenyl R moieties. Additionally, **19a** and **20a** bearing a simple phenyl and *o*-chlorophenyl R moieties displayed slightly better activity than juglone but were less active than the standard BEZ-235. These observations highlight the role of a particular moiety or a substituent in attaining the better and selective activity.

## Discussion

There is a large collection of synthetic as well as natural product derived drugs available for the treatment of various ailments, but the treatment is often unsatisfactory because of their severe side effects and drug resistance. This forms the basis for the synthesis of newer and safer drugs. The biological importance of quinones is reflected from the drugs derived from them which include doxorubicin, siantopin etc. Based on the documented anticancer activity of quinones in general and juglone in particular, we designed the synthesis of it analogs for improved and selective cytotoxic activity. Juglone is known to exhibit a broad spectrum cytotoxic activity against various cancer cell lines. In addition to this, it highly lipophilic and has therefore limited permeability across biomembranes which may lower its bioavailability upon oral administration. In order to overcome these properties, it becomes necessary to incorporate certain hydrogen bond donors in the form of heterocyclic ring systems to improve its solubility and bioavailability for better drug-likeness.

In view of the interesting anticancer activity of juglone, click chemistry inspired approach (Huisgen 3 + 2 cycloaddition) involving union of terminal alkynes with organic azides has been taken up for the synthesis of regiospecific novel OH linked triazolyl derivatives of juglone in excellent yields. The target compounds (**15a** to **20a**) were synthesized as in appreciable yields as highlighted in Scheme 1.

**16a** bearing *o*-cyanophenyl R moiety exhibited the IC_50_ of 4.67 and 7.94 μM against A549 and NCI-H322 cells respectively, while as, **15a** with *o*-nitrophenyl R moiety exhibited the IC_50_ of 4.72 and 8.90 μM against A549 and NCI-H322 cells respectively. In addition to this, **17a**, **17b** and **17c** with *o*, *m* and *p*-methoxyphenyl R moieties respectively were weakly active towards the tested cancer cell lines. **18a**, **18b** and **18c** bearing *o*, *m* and *p*-bromophenyl R moieties also exhibited less activity than juglone.

The results of this work signify that the compounds carrying an electron withdrawing groups (–NO_2_ and –CN) in the R moieties displayed better cytotoxicity than juglone, while as, the analogs with electron donating functionalities (–OMe and –Br) in the R moieties were weakly cytotoxic against both the lung cancer cell lines. Additionally, analogs with simple phenyl and *o*-chlorophenyl R moieties had little effect on the activity of the parent molecule (juglone). The results of this work reveal the effect of a particular substituent and its position on the cytotoxic activity profile of juglone. However, in vivo studies on **15a** and **16a**, the most active analogs, are warranted to investigate the exact mechanisms of action responsible for their cytotoxicity.

## Conclusions

The current study is the first report involving the synthesis of triazolyl analogs of juglone and the evaluation their in vitro cytotoxic activity using MTT assay. In this study, we have demonstrated the cytotoxic activity of a diverse library of novel triazolyl analogs of juglone prepared through Cu(I) catalyzed click chemistry approach (Huisgen 3 + 2 cycloaddition) and characterized by the analysis spectral and analytical data. Among the synthesized analogs, **15a** and **16a** were identified as highly active and selective cytotoxic agents against NCI-H322 and A549 human cancer cell lines. Both these derivatives exhibited better biological activity than BEZ-235 against both the lung cancer cell lines. So far as the SAR is concerned, electron withdrawing substituents at the ortho position of R moiety of triazoles seem to be essential for attaining the better activity.
